# Prediction of Geometric Characteristics of Melt Track Based on Direct Laser Deposition Using M-SVR Algorithm

**DOI:** 10.3390/ma14237221

**Published:** 2021-11-26

**Authors:** Xiyi Chen, Muzheng Xiao, Dawei Kang, Yuxin Sang, Zhijing Zhang, Xin Jin

**Affiliations:** School of Mechanical Engineering, Beijing Institute of Technology, Beijing 100081, China; 3120190306@bit.edu.cn (X.C.); 3220190200@bit.edu.cn (D.K.); 18810209227@163.com (Y.S.); zhzhj@bit.edu.cn (Z.Z.); goldking@bit.edu.cn (X.J.)

**Keywords:** melt track, direct laser metal deposition, multi-output support vector regression, orthogonal experimental design

## Abstract

Geometric characteristics provide an important means for characterization of the quality of direct laser deposition. Therefore, improving the accuracy of a prediction model is helpful for improving deposition efficiency and quality. The three main input variables are laser power, scanning speed, and powder-feeding rate, while the width and height of the melt track are used as outputs. By applying a multi-output support vector regression (M-SVR) model based on a radial basis function (RBF), a non-linear model for predicting the geometric features of the melt track is developed. An orthogonal experimental design is used to conduct the experiments, the results of which are chosen randomly as training and testing data sets. On the one hand, compared with single-output support vector regression (S-SVR) modeling, this method reduces the root mean square error of height prediction by 22%, with faster training speed and higher prediction accuracy. On the other hand, compared with a backpropagation (BP) neural network, the average absolute error in width is reduced by 5.5%, with smaller average absolute error and better generalization performance. Therefore, the established model can provide a reference to select direct laser deposition parameters precisely and can improve the deposition efficiency and quality.

## 1. Introduction

Due to the rapid development of the manufacturing industry, traditional processes have become unable to meet higher requirements for product accuracy, variety, and complexity. Therefore, advanced material-adding manufacturing methods have developed rapidly in recent years, especially direct laser deposition, a high-tech process that uses high-energy lasers to melt and deposit metal powder into three-dimensional parts [1]. It has attracted attention in the automobile, power plant, and aerospace industries [2], considering the associated good part performance, high manufacturing flexibility, short production cycle, and low cost [3]. However, the stability of product quality and the repeatability of manufacturing processes are not high, which seriously restricts its development.

As direct laser deposition involves complex interactions between a laser beam, metal powder, substrate material, and process gas [4], there are many uncertainties. To obtain an ideal deposition layer, numerous process tests are required, which greatly increase the required time and cost. The geometric characteristics during the deposition process are the main factors when measuring whether or not the formation conditions are reasonable. The relationship between the process parameters and the geometric characteristics of the melt track is nonlinear and difficult to be described by a simple mathematical model, so it is of the highest importance to establish an accurate and efficient prediction model using a small amount of experimental data to quickly obtain an ideal deposit.

Prediction of the geometric characteristics of a melt track is a multivariate non-linear regression problem [5]. In the literature, research efforts have focused on predicting the geometric characteristics of metal deposition using statistical and data mining methods based on prior experimental work. Sun [6] established a mathematical model by using a central composite design and response surface method. Through an analysis of variance (ANOVA) test of the established model, the relationships between the process parameters and output response and the interaction between the parameters were analyzed and discussed in detail. Mohammad [7] used a linear regression analysis to research the empirical–statistical relationship between key parameters and the geometric characteristics of the melt path.

Zhang [8] chose the multiple linear regression model to find the influence rule of the main parameters on the size of single-track 30CrNi_2_MoVA steel cladding. The significant factors affecting the width and height of the melt channel and the corresponding regression equation were determined. Davim [9] established a model using multiple regression analysis (MRA) between processing parameters and the form of single-cladding layer (clad height, clad width, and depth penetration into the substrate); however, the laser cladding process is complicated and nonlinear, so it is difficult to use only an equation to describe this correlation.

Machine learning is a widely used powerful tool that is suitable for determining the non-linear correlations between input variables and output results. In recent years, neural networks have been widely used for predicting direct laser deposition. Xu [10] designed a learning algorithm based on an evolutionary neural network and established a prediction model between the melt dilution rate, cladding width and height, and process parameters. The model overcomes the local optima problem often observed with neural networks. Acherjee [11] developed an artificial neural network (ANN) model to predict the quality characteristics of thermoplastic laser welding. In addition, the prediction results of the ANN were compared with those of multiple regression analysis models, demonstrating that its prediction was better than that of the regression model. Mondal [12] studied multi-objective optimization in the process of CO_2_ laser cladding with the width and depth of the cladding layer as the performance indices. Many experiments were carried out using the ANN backpropagation method, and the relationship between the process and response variables was established. Fabrizia [13] developed an ANN-based machine learning method to determine the correlation between the laser cladding process parameters and the size parameters of a single channel on a 2024 aluminum alloy plate. This method was adjusted several times in order to achieve a high-precision model. Yin [14] proposed a backpropagation (BP) ANN model to obtain the mathematical relationship between the optimization goals and process parameters and applied a genetic algorithm to optimize the parameters. In order to speed up the convergence and avoid local minimum of the conditional ANN, Zhong [15] inducted genetic algorithm simulated annealing (GASA) based on the random global optimization into the network training. Meanwhile, the gray correlation model (GCM) was used as a pre-processing tool to simplify the original networks based on obtaining the main influence elements of network inputs. On this basis, a genetic algorithm was applied to optimize the parameters. Although neural networks have been widely used, due to their ability to process multiple and non-linear information in parallel, the problem of poor generalization ability occurs for a small sampling size, such as the one used in this paper.

Through statistical learning theory analysis, it can be seen that the neural network adopted the strategy of minimizing empirical risk, and the data volume and dimensions affected its regression performance. On the other hand, support vector machines(SVM) adopts the strategy of minimizing structural risk; thus, integrating empirical and confidence risk and achieving global optimization more easily. SVM can be used for classification and regression, and has been successfully applied in many practical fields, proving that its generalization ability is better than that of a neural network. As an extension of SVM, the support vector regression (SVR) method can be considered very suitable for direct laser deposition geometric characteristics prediction, and can address the problems of insufficient training data, non-linearity, and strong output coupling, as was the case in this study. To research the transition temperature of NdBa2Cu3O7-u03b4(NBCO) thin films prepared by pulsed-laser deposition, Xiao [16] proposed a prediction model of pressure, temperature, and laser energy on transition temperature based on SVR. The result showed that the average absolute error of SVR was less than that of multiple non-linear regression. Yang [17] established an SVR prediction model for the WC volume fraction under different process parameters, which had a smaller prediction error and stronger generalization performance than an ANN model. Ye [18] took underwater wet shallow-water flux-cored arc welding as a research object, and used SVR to improve the modeling accuracy and prediction speed. Tao [19] screened out the process parameters that had a strong impact on multiple quality characteristics based on signal-to-noise (S/N) and analysis of variance, established four SVR models to predict each quality characteristic and, finally, proved that this method has high prediction accuracy. Yao [20] compared the prediction performance of SVR models based on different kernel functions, and found that the model based on a radial basis function (RBF) was more suitable for predicting the geometric characteristics of the deposited melt channel. However, it should be noted that SVR mainly addresses the single-output problem and often uses the construction of multiple SVR models to solve a multi-output problem. This means unequal treatment of each sample point, which not only slows the training speed but also affects the prediction accuracy [21].

By analyzing the correlation of geometric characteristics in direct laser deposition, the output parameters are shown not to be independent. Single-output support vector regression (S-SVR) ignores the relationships between different output parameters, thus affecting the prediction accuracy. Therefore, this paper proposes a method for predicting laser-deposition geometric characteristics based on multi-output support vector regression (M-SVR). By comparing the predicted results to those of neural network and S-SVR models, the fitting accuracy and generalization ability of the model are detailed.

## 2. Experimental Procedure

### 2.1. Experimental Materials

For the test, 4 × φ150 mm of 316-L stainless steel was selected as the base material. Before laser deposition, the base material was sandblasted and cleaned with alcohol and acetone. Products using 316-L stainless steel powder as deposition material have been widely used in the nuclear, biomedicine, and aerospace industries, as 316-L stainless steel powder material is cheap, and has excellent corrosion and oxidation resistance and biocompatibility. The particle size was 15–53 μm, and the specific chemical composition is shown in Table 1. Due to the need to maintain a stable powder feeding rate [22], the powder was dried at 120 °C for 5 h before the experiment.

### 2.2. Experimental Setup

**Figure 1:**
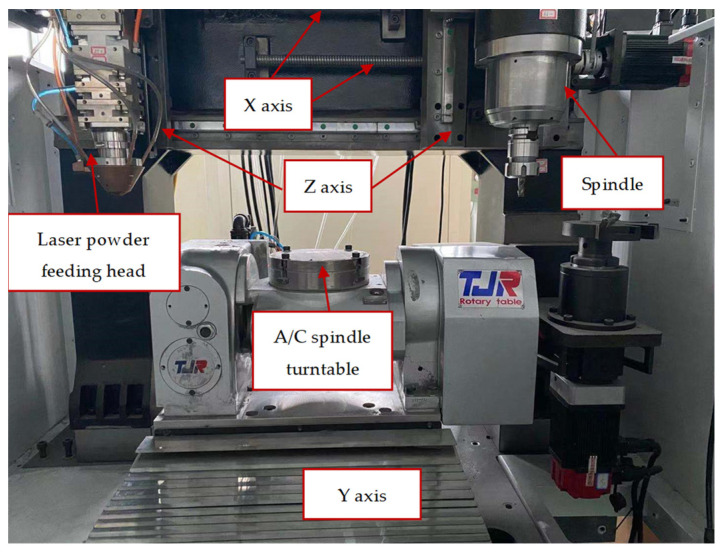
Schematic diagram of experimental setup.

A 2-kW laser with a wavelength of 980 nm was used to deposit 316 L onto the substrate. The laser cladding head was built on a five-axis, material-increasing-and-decreasing compound machining center driven by five-axis motion, in order to increase the amount of material. Figure 1 shows the schematic diagram of an experimental setup. The powder material was injected into a discrete coaxial nozzle that can work with the laser optical fiber of LDM2000-60 (Deloitte, Ningbo, China), in order to perform optical coaxial powder-feeding deposition. The powder was delivered to the nozzles by means of argon gas through four single-cylinder powder feeders.

**Figure 2:**
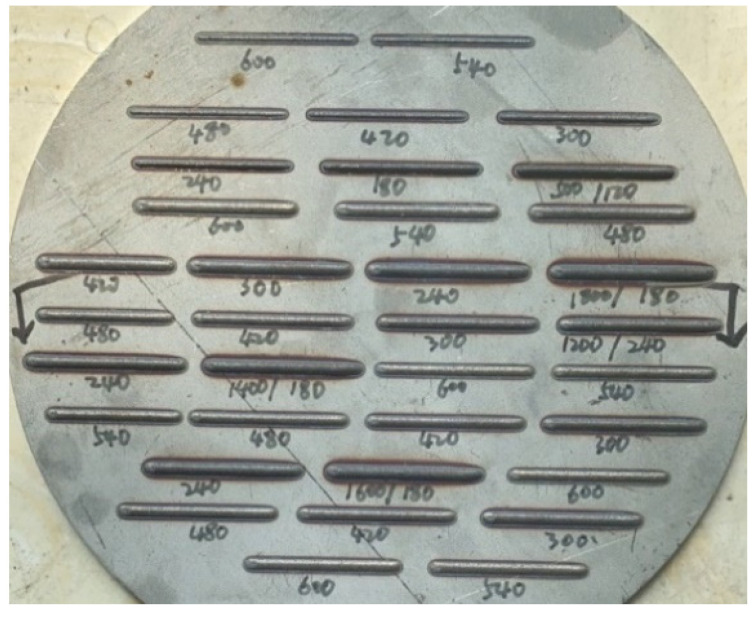
Appearance of the melt track.

**Figure 3:**
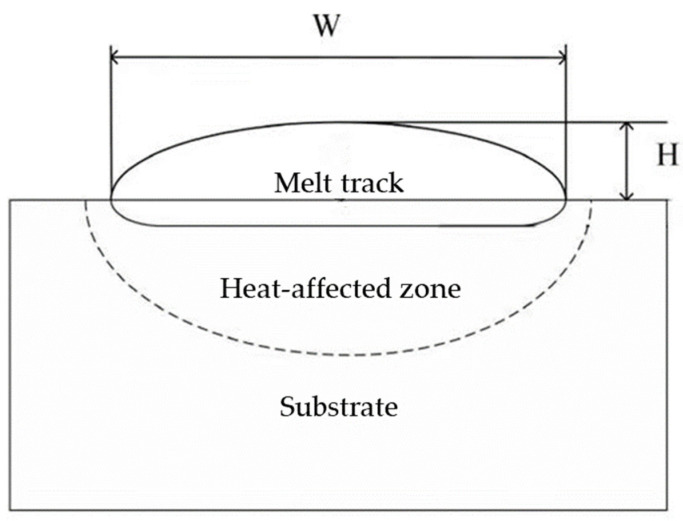
Schematic diagram of the melt channel cross section.

**Figure 4:**
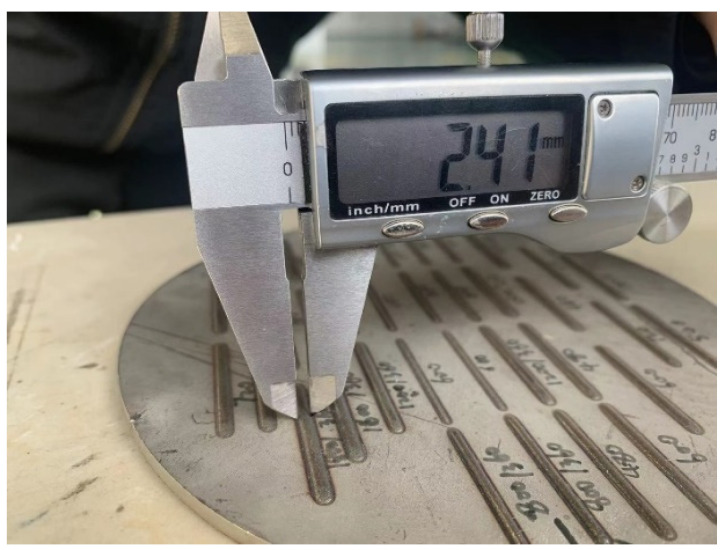
Width measurement measurements using vernier caliper.

**Figure 5:**
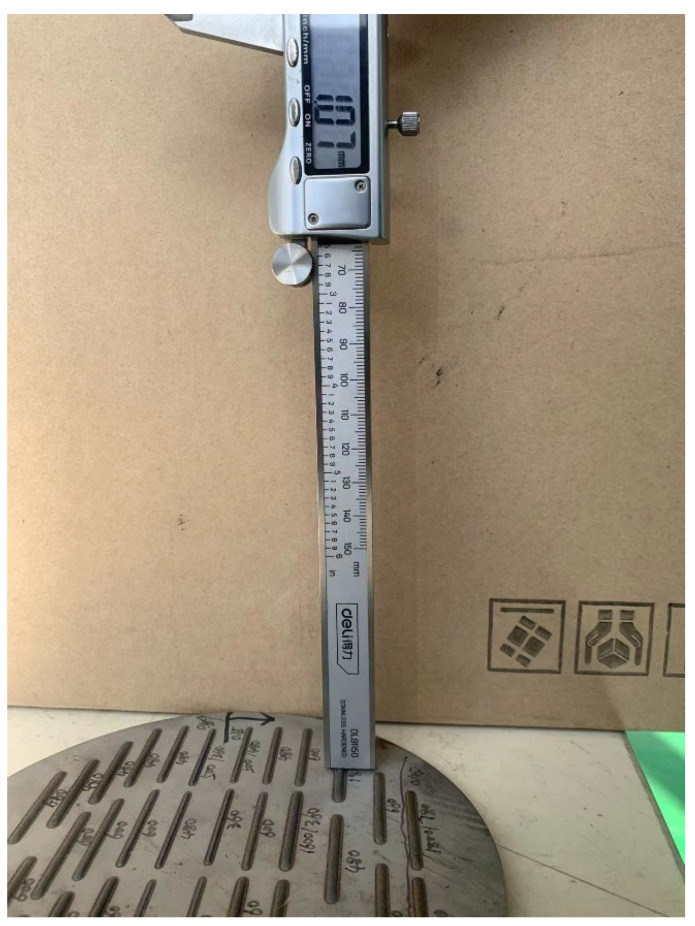
Height measurement measurements using vernier caliper.

The literature [19] has indicated the three factors that have the greatest impact on the quality of laser cladding: laser power, scanning rate, and powder feeding rate. Therefore, these factors were taken as input variables in this paper. The geometric characteristics were mainly characterized by the width and length of the melt track. The width is the lateral distance of the deposited part on the substrate plane, while the height is the distance of the deposited part above the substrate plane. Using vernier caliper to measure the height and width of the melt track, in order to avoid measurement errors, the three positions are measured and their average value is taken, and the deviation of the size does not exceed 0.03 mm. The parameters of this vernier caliper are shown in Table 2. The appearance of the melt track is shown in Figure 2, and the cross section of the single-layer melt channel is shown in Figure 3. Height and width measurement using vernier caliper are shown in Figure 4 and Figure 5.

### 2.3. Experimental Data

The multi-output support vector regression (M-SVR) model took laser power, powder-feeding rate, and scanning speed as inputs, and deposition height and width as outputs. The data required for SVR modeling should fully reflect the role of each parameter, but it is unrealistic to conduct a large number of deposition experiments to cover all parameter combinations. Orthogonal tests only require part of the full factor design combination, such that a few combinations can be used to evaluate more test factors and the most reliable conclusion can be obtained [23]. A total of 3^3^ = 27 sets of tests were required, and the test results are shown in Table 3. It shows the result of the geometric characteristic of clad width (W) and clad height (H), as a function of the processing parameters (laser power (P), scanning velocity ($V_{s}$), and powder mass flow rate ($V_{f}$).

## 3. Principles and Methods

### 3.1. Basic Principles of the Multi-Output Support Vector Regression Algorithm

Support vector regression belongs to black-box modeling, and fits the relationship between input and output by analyzing known sample data. A set training sample is used to construct the regression function [24]:

(1) $$F\left(x\right)=\Phi {\left(x\right)}^{T}\cdot W+B$$

where F(x) is the *N*-dimensional output function; the nonlinear mapping $\Phi {\left(x\right)}^{T}$ maps the data x to a high-dimensional feature space; the non-linear mapping maps the data to a high-dimensional feature space; and *W* and *B* are the weight vector and the bias, respectively. Therefore, the multiple regression problem to be solved is to find the regression quantity for each output $\omega^{j}$ and $b^{j}$, when the objective function L(W,B) is:

(2) $$Min\;L\left(W,B\right)=\frac{1}{2}\overset{l}{\underset{i=1}{\sum }}\|W^{j}\|{}^{2}+C\overset{l}{\underset{i=1}{\sum }}L\left(u_{i}\right)$$

(3) $$L\left(u_{i}\right)=\{\begin{array}{c} \begin{array}{c} 0\;\;\;\;\;\;,\;\|u_{i}\|<\epsilon \;\;\;\\ {\left(\|u_{i}\|-\epsilon \right)}^{2}\;,\;\|u_{i}\|>\epsilon \; \end{array} \end{array}$$

where $L\left(u_{i}\right)\;$represents the loss function (ϵ-insensitive function) defined on a hyperball; *i* represents the number of samples, *j* represents the dimensionality of the output; l represents the total number of samples; and $u_{i}=\|e_{i}\|=\sqrt{e_{i}e_{i}^{T}},e_{i}=y_{i}-\Phi {\left(x\right)}^{T}\cdot W-B$, where $y_{i}$ is the actual output value of the *i*-th sample. When ϵ=0, the problem becomes a least-squares regression for each output component. When ϵ≠0, the fitting effect of other output components will be taken into account when solving the regression quantities $\;\omega^{j}$ and $b^{j}$ of each output function, such that the solution obtained will be the best solution for the overall fitting.

Based on the objective function and the constraints, the following Lagrange functions can be obtained, where $\alpha_{i}$ is Lagrange multiplier:

(4) $$L\left(W,B\right)=\frac{1}{2}\sum_{i=1}^{l}\|W^{j}\|{}^{2}+C\sum_{i=1}^{l}L\left(u_{i}\right)-\sum_{i=1}^{l}\alpha_{i}\left(u_{i}^{2}-\|y_{i}-\Phi {\left(x\right)}^{T}\cdot W-{B\|}^{2}\right)$$

Let $\frac{\partial L}{\partial W}=\frac{\partial L}{\partial B}=0$. Then the following equation is obtained:

(5) $$W^{j}-\sum_{i=1}^{l}\varphi \left(x_{i}\right)\alpha_{i}\left(y_{ij}-\varphi^{T}\left(x_{i}\right)w^{j}-b^{j}\right)=0$$

(6) $$-\sum_{i=1}^{l}\alpha_{i}\left(y_{ij}-\varphi^{T}\left(x_{i}\right)w^{j}-b^{j}\right)=0$$

By introducing the core function K=$\varphi {\left(x_{i}\right)}^{T}\varphi \left(x_{j}\right)$ and $W^{j}=\overset{l}{\underset{i=1}{\sum }}\beta^{j}$, the following matrices can be obtained:

(7) $$\left[\begin{array}{cc} K+D_{\alpha }^{-1} & 1\\ \alpha^{T}K & 1^{T}\alpha \end{array}\right]\left[\begin{array}{c} \beta^{j}\\ b^{j} \end{array}\right]=\left[\begin{array}{c} y^{j}\\ \alpha^{T}y^{j} \end{array}\right],j=1,\cdots ,N$$

where $D_{\alpha }=diag\left(\alpha_{1},\alpha_{2},\ldots ,\alpha_{\;N}\right)$. Therefore, solving $\omega^{j}$ and $b^{j}$ has evolved into solving $\beta^{j}$ and $b^{j}$.

### 3.2. Basic Principles of Backpropagation Neural Network

**Figure 6:**
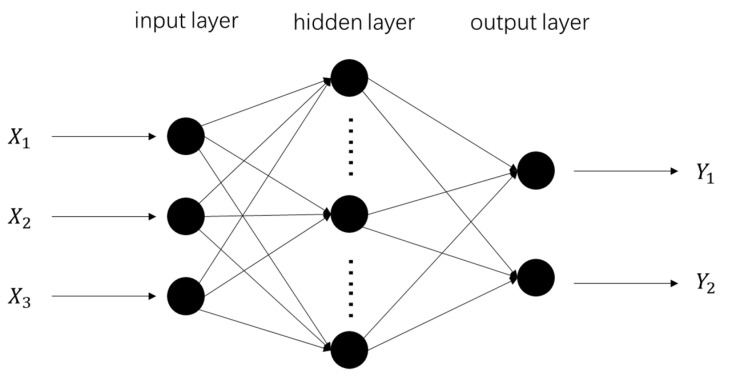
Backpropagation neural network used for predicting geometric characteristics.

Backpropagation is a feed-forward neural network trained by Rumelhart in 1986, constructed according to an error backpropagation algorithm that consists of input, hidden, and output layers [25]. The structure of a neuron is shown in Figure 6. Each neuron is represented by a node, and the input, hidden, and output layers are all composed of nodes. Each layer of nodes is transferred to the next layer by weights and biases, consisting of forward and backpropagation calculations. When forward propagation occurs, the input layer is transferred to the output layer through the hidden layer. If the output layer cannot get the desired result, it propagates back, returns the error signal along the original path, and finally minimizes the error signal by modifying the weights of each neuron. The number of hidden layer neurons is calculated using the following formula:

(8) $$c=\sqrt{k+p}+a$$

where *k* is the number of hidden layers, *p* is the number of neurons in the input layer, *n* is the number of neurons in the output layer, and *a* is any integer from 1 to 10. In this article, *k* is 3, *p* is 2, and *a* is 3; so, *i* is 5.

## 4. Result and Discussion

### 4.1. Prediction Model Establishment

The literature [20] has shown that SVR based on a radial basis function (RBF) is more suitable for predicting melt track size. The experimental results in Table 3 were randomly divided: 22 tests were randomly selected as training samples, and the other 5 tests were used as testing samples. Due to the large variation in the range of different process parameters, these data needed to be normalized and de-normalized before model training.

**Figure 7:**
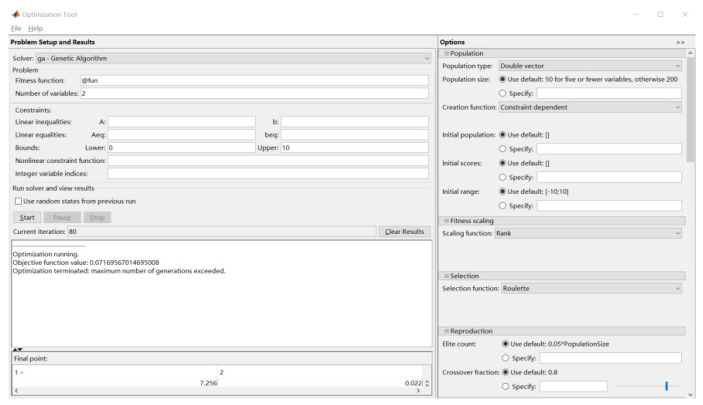
Genetic algorithm toolbox.

**Figure 8:**
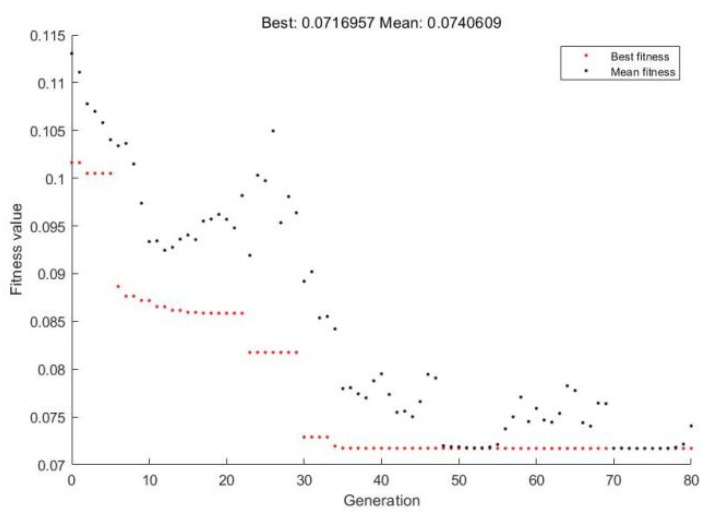
Iteration results.

To build a regression model using RBF-based M-SVR, it is important to choose two hyperparameters: the regularization factor C and the kernel function σ. The regularization parameter C mainly affects the robustness of the regression model, with a small value of C indicating a small penalty for empirical error, which increases the training error, while a large value of C leads to poor generalization of the SVR model; the kernel function σ mainly affects the complexity of the high-dimensional feature space distribution, with over- fitting and under-fitting when σ is not appropriate. For this reason, the genetic algorithm is used in this paper to find the optimal parameters. The regularization parameters C and kernel function parameters σ were encoded for the regularization parameters, the fitness function is the root mean square error of the error, the control parameters are set as the initial population size of 50, the maximum number of iterations is 80, the crossover rate is 0.8, the variation rate is 0.2, etc. The settings in the MATLAB genetic algorithm toolbox are shown in Figure 7. The iterative results are shown in Figure 8, where the red and black dots are the fitness value of the best individual in each generation and the average of the fitness value of all individuals in that generation, respectively, and the fitness value is basically constant after 40 generations, which can be combined with Figure 7 to show that the operation results are C = 7.2 and σ = 0.02.

### 4.2. Performance Evaluation

The M-SVR model was evaluated for accuracy and generalization performance.

The root mean square error (*RMSE*) and correlation coefficient ($R^{2}$) of M-SVR and S-SVR were evaluated using the five tests of testing samples. In addition, another five tests of experiments designed within the specified process parameters were used as new samples, in order to test the generalization performance. In this group of data, the root mean square error (*RMSE*) and mean absolute error (MAE) of M-SVR and BP were compared. For all the following formulas: n is the number of samples, $y_{i}$ is the true value, ${\hat{y}}_{i}$ is the predicted value of the i sample, and $\bar{y}$ is the average of the true values.

(9) $$RMSE=\frac{1}{n}\sqrt{\overset{n}{\underset{i=1}{\sum }}{\left(y_{i}-{\hat{y}}_{i}\right)}^{2}}$$

(10) $$R^{2}=\frac{\sum_{i=1}^{n}\left({\hat{y}}_{i}-\bar{y}\right)}{\sum_{i=1}^{n}{\left(y_{i}-\bar{y}\right)}^{2}}$$

(11) $$RAE=\frac{1}{n}\overset{n}{\underset{i=1}{\sum }}\left|{\hat{y}}_{i}-y_{i}\right|$$

### 4.3. Prediction Accuracy Analysis

Model fitting accuracy is a very important indicator, especially considering the complicated working conditions of direct laser deposition. In this paper, M-SVR and S-SVR models based on an RBF were established, using the same hyper-parameters. The prediction model is obtained by inputting training samples and evaluated with a test sample set. The overall *RMSE* was calculated $\left(RMSE_{1}+RMSE_{2}+\ldots RMSE_{N}\right)/N$ (*N* is the dimension of the output). For $R^{2}\;$use the same method, $R^{2}=\left(R_{1}^{2}+R_{2}^{2}+\ldots R_{N}^{2}\right)/N$. These results are shown in Table 4.

From the table data, it can be seen that the fitting correlation coefficients of M-SVR and S-SVR were the same, but the root mean square error of M-SVR was 0.07, i.e., much smaller than that of SVR. Therefore, the prediction accuracy of M-SVR was better than that of S-SVR.

### 4.4. Model Validation

**Figure 9:**
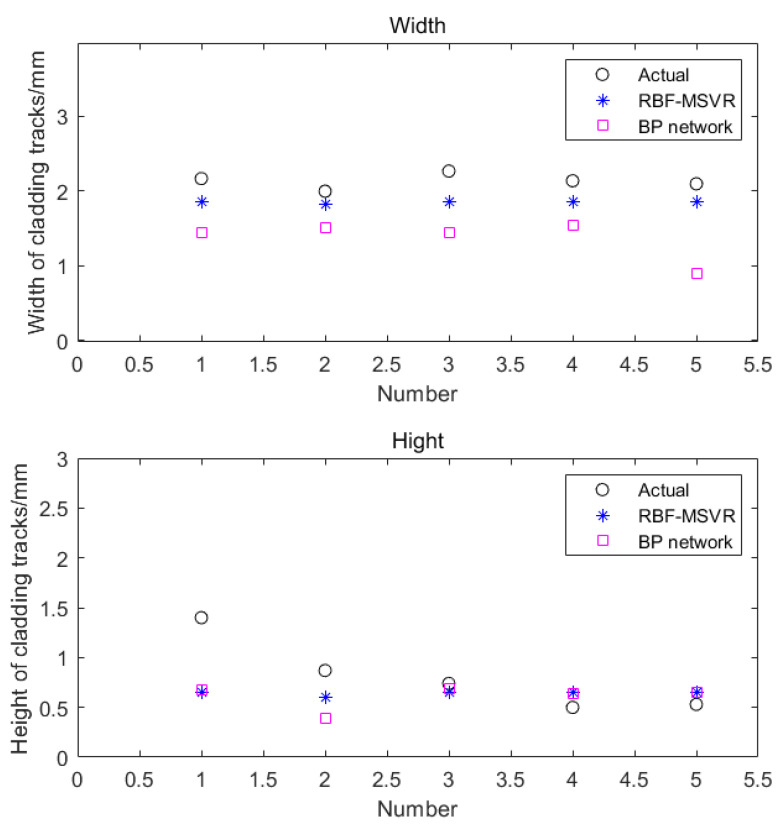
Comparison between actual value and predicted value of weld bead size.

The above generalization performance testing samples were input into the trained model, and the predicted and real values of the height and width were compared to assess the generalization ability of the two models. The process parameter scheme is shown in Table 5. A comparison of the real weld bead width and height values with those predicted by the M-SVR and BP neural network, for the generalization performance test, is shown in Figure 9.

From the scatter diagram, it can be seen that M-SVR performed better than the BP neural network, in terms of predicting the width of the melt channel. However, the accuracy of the two is basically the same in height prediction. To enhance our understanding, the above prediction results were analyzed; that is, the *RMSE* and MAE of the different models were calculated. The analysis results are shown in Table 6. M-SVR was far better than the BP neural network for predicting the width of the melt channel. The prediction value of the width and height each differed from the true value by 0.05 mm, compared with the BP neural network. The prediction results of M-SVR increased by 0.10 and 0.01 mm, respectively. The improvement in height is negligible, but, in general, it improves the generalization performance of M-SVR which was better for predicting of the melt channel size.

## 5. Conclusions

To predict the melt channel size in direct laser deposition, a melt-channel size prediction method based on an M-SVR algorithm was proposed, by utilizing the characteristics of small samples and by assessing the correlations between inputs and outputs. An M-SVR prediction model, with three input process parameters (laser power, powder-feeding rate, and scanning speed) and melt channel width and height as outputs, was established. This model was compared with SVR and BP neural network models in terms of model accuracy and generalization performance. The prediction accuracy of S-SVR and M-SVR was assessed on a testing sample set, and the results indicated that the M-SVR model performed better. The generalization performance on the training and testing sample sets of M-SVR and the BP neural network showed that the mean absolute errors of M-SVR were 0.05 and 0.05 mm, respectively, which were smaller than that of the BP neural network, comprising an improvement in size prediction accuracy by 0.10 and 0.01 mm (negligible in height), respectively. In summary, the proposed M-SVR model established a good mapping relationship between the process parameters and the geometric features in direct laser deposition and showed good generalization performance.

## Figures and Tables

**Table 1:** Chemical composition (mass fraction) of 316 L stainless steel powder (%).

Cr	Ni	Mo	Si	Mn	O	S	C	Fe
17.92	12.04	2.42	0.52	0.051	0.0451	0.010	0.0095	allowance

**Table 2:** Parameters of vernier caliper.

Type	Measurement Uncertainty	Measurement Range	Resolution
Deloitte: DL91150	±0.03 mm	150 mm	0.01 mm

**Table 3:** Orthogonal deposition test results.

Test Number	P/W	$V_{s}/\left(mm\cdot min^{-1}\right)$	$V_{f}/\left(g\cdot min^{-1}\right)$	W/mm	H/mm
1	800	360	0.25	1.60	0.62
2	800	360	0.35	1.54	0.80
3	800	360	0.45	1.60	0.94
4	800	480	0.25	1.49	0.45
5	800	480	0.35	1.44	0.65
6	800	480	0.45	1.50	0.83
7	800	540	0.25	1.46	0.35
8	800	600	0.35	1.36	0.56
9	800	600	0.45	1.40	0.64
10	1200	360	0.25	2.05	0.72
11	1200	360	0.35	2.00	0.87
12	1200	360	0.45	2.14	1.10
13	1200	480	0.25	1.81	0.47
14	1200	480	0.35	1.90	0.71
15	1200	480	0.45	2.00	0.94
16	1200	600	0.25	1.76	0.35
17	1200	600	0.35	1.78	0.62
18	1200	600	0.45	1.80	0.73
19	1800	360	0.25	2.45	0.53
20	1800	360	0.35	2.39	0.95
21	1800	360	0.45	2.42	1.05
22	1800	480	0.25	2.20	0.40
23	1800	480	0.35	2.21	0.70
24	1800	480	0.45	2.28	0.78
25	1800	600	0.25	2.00	0.32
26	1800	600	0.35	2.15	0.58
27	1800	600	0.45	2.20	0.65

**Table 4:** Fitting accuracy analysis of different models.

Model	*RMSE*	*R* ^2^
M-SVR	0.07	0.92
S-SVR	0.54	0.92

**Table 5:** Generalized performance test set.

Test Number	P/W	$V_{s}/\left(mm\cdot min^{-1}\right)$	$V_{f}/\left(g\cdot min^{-1}\right)$	W/mm	H/mm
1	1000	180	0.25	2.17	1.40
2	1200	600	0.35	2.00	0.87
3	1400	240	0.25	2.27	0.74
4	1600	420	0.25	2.14	0.50
5	1800	660	0.35	2.10	0.53

**Table 6:** Error analysis of prediction results of different models.

Model	Prediction of Track Width(mm)		Prediction of Track Height (mm)	
	*RMSE*	MAE	*RMSE*	MAE
BP neural network	0.15	0.15	0.05	0.06
M-SVR	0.02	0.05	0.05	0.05

## Data Availability

The data presented in this study are available from the corresponding author upon reasonable request.
